# Emergency treatment of a ruptured huge omphalocele by simple suture of its membrane

**DOI:** 10.1186/1750-1164-6-2

**Published:** 2012-02-12

**Authors:** Gamedzi Komlatsè Akakpo-Numado, Komla Gnassingbe, Missoki Azanledji Boume, Kodjo Abossisso Sakiye, Komlan Mihluedo-Agbolan, Komlan Attipou, Hubert Tekou

**Affiliations:** 1 Department of pediatric surgery, Tokoin Teaching Hospital, BP: 57, Lomé, Togo; 2 Department of visceral surgery, Tokoin Teaching Hospital, BP: 57, Lomé, Togo; 3 Department of pediatric surgery, Tokoin Teaching Hospital, 08 BP 80025, Lomé 8, TOGO

**Keywords:** Ruptured omphalocele, Huge omphalocele, Grob's method, Developing countries

## Abstract

**Background:**

The rupture of a huge omphalocele is an emergency that threatens the newborn baby's life. It constitutes a therapeutical concern in the absence of prosthesis especially in developing countries.

**Methods:**

We are reporting herein the case of a newborn baby that we managed in emergency successfully thanks to a simple treatment.

**Results:**

It was a huge omphalocele, ruptured during delivery, in a male newborn baby. We conducted a simple and conservative surgical treatment without prosthesis, which consisted of reconstruction of the omphalocele's membrane by closing it with absorbable suture materials. The suture of the omphalocele's membrane was followed by treatment with the Grob's method. This treatment saved the newborn baby's life. The total skinning was obtained after 3 months.

**Conclusions:**

In case of rupture of huge omphalocele in absence of prosthesis, it is better to suture the membrane, and continue the treatment according to the Grob's method; the residual disembowelment can be repaired later.

## Background

The management of omphalocele has been improved, due to advances that occurred in pediatric anesthesia and resuscitation [1-3]. However, developing countries remained far from these advances not only because of the limited conditions of anesthesia and resuscitation [4], but also because of the unavailability of prosthetic materials required for the surgical treatment of huge omphalocele. Thus, the non surgical treatment according to Grob is often done [4], followed by a surgical repair of the residual disembowelment. In these conditions, the situation becomes difficult when one faces a ruptured huge omphalocele (type II of Aïtken), because the Grob's method cannot longer be used.

Hereby, we are reporting the case of a huge omphalocele, ruptured during delivery and treated surgically with a simple technique without the use of prosthetic materials.

## Case presentation

**Figure 1:**
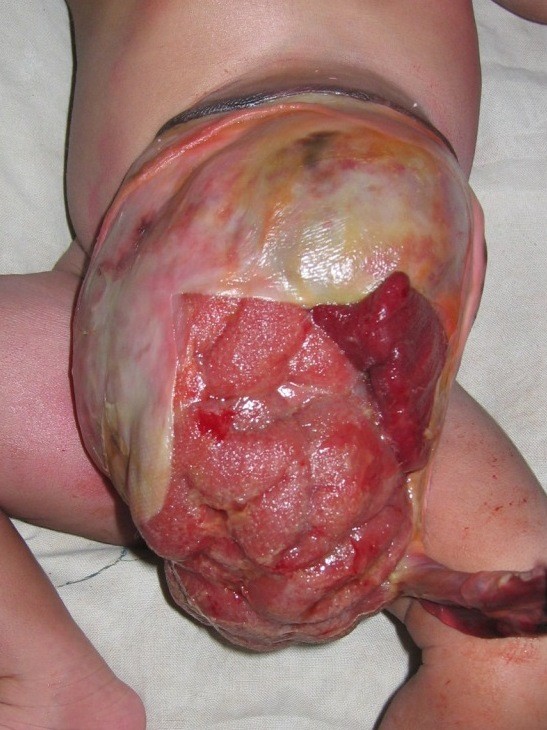
**Ruptured omphalocele showing a part of the liver and small bowel**.

**Figure 2:**
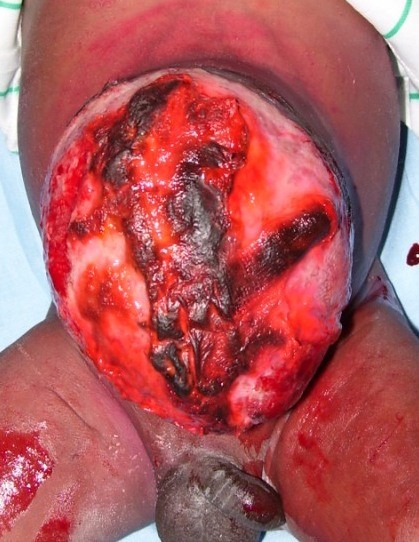
**Aspect of the omphalocele 15 days after surgical repair of the membrane, and its staining with aqueous eosin**.

**Figure 3:**
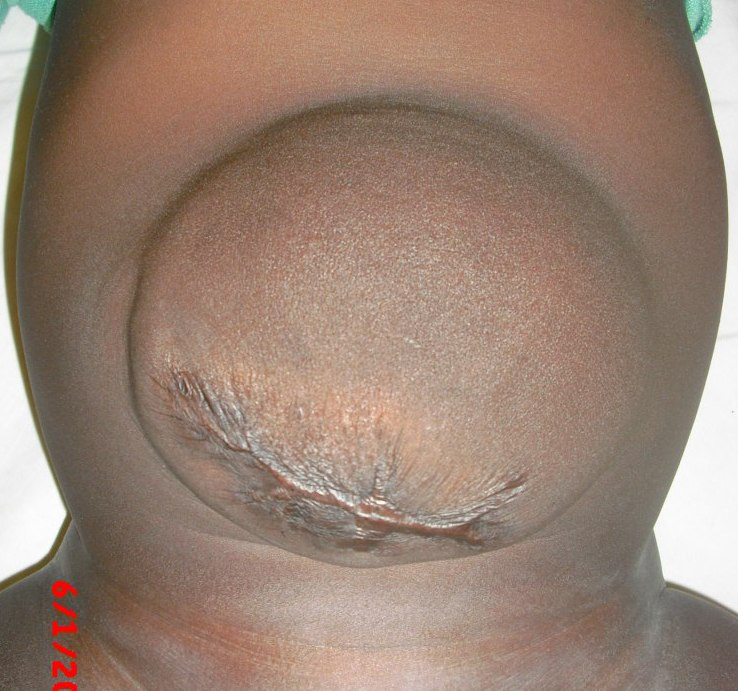
**Residual disembowelment after total skinning of the membrane**.

It was a male newborn baby, born on December 25th, 2007 in a clinic and transferred immediately to the Tokoin Teaching Hospital for abdominal defect with evisceration. He was born by spontaneous vaginal delivery following a normal term pregnancy. No ultrasound (US) scan was done during pregnancy. There was no family history of malformation. He was not resuscitated at birth; his birth weight was 4.1 Kg, and his height was 53 cm. He had no macroglossia. On abdominal examination there was an omphalocele type II of Aïtken, measuring 14 cm of neck diameter, and 10 cm of average diameter. The umbilical cord was located at the top. There was a vertical cracking of the omphalocele's membrane in Y-shaped form, from the implantation of the umbilical cord to the neck of the omphalocele, exposing a part of the anterior face of the liver, and other viscera (Figure 1). There was no other obvious malformation associated. The newborn baby was admitted into the theater and after examination under general anesthesia, we noticed that the content of the omphalocele included all the small bowel, a part of the colon, stomach and the lower part of the liver. There was no Meckel's diverticulum. Left and right colon were not joined to the posterior wall of the abdomen. The volume of viscera in the omphalocele's sack was so important that their reintegration into the abdomen after resection of the sack was impossible. No prosthesis was available. Therefore, we reintroduced viscera in the abdomen and the omphalocele, and then we conducted a suture of the membrane with absorbable suture materials (breaded polyglycolic acid N° 0). The postoperative period was uneventful. The treatment was followed by staining of the membrane with aqueous eosin according to Grob's method. The omphalocele was always protected by a bandage. The bandage was changed every 2 days, and prophylactic therapy by antibiotics (Amoxicillin and clavulanic acid) was done for two weeks. The skinning of the membrane occurred progressively without tearing or suppuration (Figure 2). The complete skinning was obtained at the end of 3 months, leaving a voluminous disembowelment (Figure 3) that should benefit later from further surgical repair.

## Discussion

The management of omphalocele aims to reintegrate the herniated viscera in the peritoneal cavity and to repair the anterior abdominal wall [5]. This reinstatement must be done without abdominal compression. So it is necessary, after reinstatement of viscera into the abdomen and before abdominal wall repair, to check whether there is any cyanosis, respiratory and circulatory disorders [2,6]. The abdominal wall's closure with tension causes respiratory and circulatory disorders, intestinal necrosis and infection leading to death [2]. In addition to the advances in pediatric anesthesia and resuscitation, the use of prosthesis has also increased the postoperative survival of children with omphalocele [7,8]. In developing countries, prosthesis are often unavailable and we often use non-surgical treatment in emergency according to the Grob's method. The Grob's method consist in staining of the omphalocele's membrane with an antiseptic until total skinning [4,6]. A bandage can be done after staining, especially for huge omphalocele as in our case. The skinning starts from the neck of the omphalocele and spreads up to the top in a centripetal way. The full skinning is obtained at about 3 months. After this, it remains a disembowelment, that requires a surgical repair later. The Grob's method has many advantages: it permits us to avoid the risks of neonatal surgery in our conditions; it doesn't need prosthetic materials and it is well indicated in developing countries. During treatment with Grob's method, the buccal feeding is continued, whereas the treatment with prosthesis requires parenteral feeding. The parenteral feeding is expensive and is not always available in most of developing countries. The Grob's method needs the integrity of the omphalocele's membrane. It is not indicated when the omphalocele's membrane is ruptured as it was the case in our patient. It is therefore difficult to manage the rupture of a huge omphalocele when prosthesis are not available. This rupture can be prevented if prenatal US scan diagnosis is done. The US scan diagnosis enable the obstetrician, to estimate the volume of the swelling and to select the less traumatic mode of delivery for the baby. In our case, prenatal US scan diagnosis was not done, and the omphalocele was discovered, ruptured at birth.

Small omphalocele (type I) can benefit from single surgical repair. Those that cannot benefit from single surgical repair can be processed according to the technique of Ladd and Gross [6]; this technique consist in cutting off the omphalocele's membrane, and then, reinstatement of viscera into the abdomen, and surgical closure of only the skin without the aponeurosis. This creates a surgical disembowelment, that should be repaired later. If the volume of viscera out of the abdomen is important, the skin closure can require lateral incision of discharge, in order to increase the volume of abdominal cavity. However, there are some risks of infection associated with these incisions of discharge, so it is better to avoid them and to choose gradual reduction using a prosthesis (silastic) [7,8]. This gradual reduction is followed later by a correct abdominal wall repair [7]. This technique greatly improves the vital prognosis of infants with omphalocele, unless there are other debilitating malformations associated with [5]. The rupture of an omphalocele is an extreme surgical emergency that cannot be managed according to Grob's method [4,6]. When the ruptured omphalocele is small, surgical repair without prosthesis may be done. When it is huge, the use of prosthesis becomes necessary [9]. It is well known that the surgical repair of these huge omphalocele without prosthesis, leads to fatal complications [2]. One can doubt on ability of healing, of a sutured membrane of omphalocele; but our experience proves the contrary. It is better, to preserve the vital prognosis of the newborn baby, by suturing the membrane in one plan with separated stitches using thick suture material (polyglycolic acid N° 0 in our case). After the reconstitution of the membrane, the treatment is continued by staining the membrane with an antiseptic (aqueous eosin in our case). The full skinning of the membrane is obtained within 2 to 3 months, and the surgical treatment of the residual disembowelment can be done later.

## Conclusions

The rupture of a huge omphalocele in developing countries should no longer be considered as a fatality. A good surgical repair of the membrane followed by its staining with antiseptic can permit to preserve vital prognosis of those children.

## Consent

Written informed consent was obtained from the patient's parents for the publication of this Case report and any accompanying images. A copy of the written consent is available for review by the Editor-in-Chief of this journal.

## Competing interests

The authors declare that they have no competing interests.

## Authors' contributions

ANGK received, operated and followed the patient; he wrote the manuscript. GK participated to the follow-up. BMA and SKA participated to the operation and the follow-up. MAK, AK and TH helped to draft the manuscript. All authors read and approved the final manuscript.
